# Association between social jetlag and chronic kidney disease among the Korean working population

**DOI:** 10.1038/s41598-023-33130-1

**Published:** 2023-04-12

**Authors:** Seong-Sik Cho, Byung Ha Chung, Hye-Eun Lee, Mo-Yeol Kang

**Affiliations:** 1 Department of Occupational and Environmental Medicine, College of Medicine, Dong-A University, Busan, Republic of Korea; 2 Division of Nephrology, Department of Internal Medicine, Seoul St. Mary’s Hospital, College of Medicine, The Catholic University of Korea, Seoul, Republic of Korea; 3 Department of Social and Preventive Medicine, Hallym University College of Medicine, Chuncheon, Republic of Korea; 4 Department of Occupational and Environmental Medicine, Seoul St. Mary’s Hospital, College of Medicine, The Catholic University of Korea, 222, Banpo-daero, Seocho-gu, Seoul, 06591 Republic of Korea

**Keywords:** Neuroscience, Nephrology, Risk factors

## Abstract

Social jetlag refers to the discrepancy between social time and the body’s internal rhythm, which can lead to unfavorable health outcomes. However, no study has directly explored the relation between social jetlag and chronic kidney disease (CKD). This study aims to investigate the relationship between social jetlag and CKD in a representative population of South Korea. This study included 8259 currently economically active Korean population in the Korea National Health and Nutrition Examination Survey. Social jetlag was calculated as the difference between the midpoint of sleep time on weekdays and free days. The estimated glomerular filtration rate (eGFR) was calculated the by using the serum creatinine value according to the Chronic Kidney Disease Epidemiology Collaboration (CKD-EPI) equation. Participants with an eGFR less than 60 ml/min/1.73 m^2^ were defined as CKD cases. The estimated glomerular filtration rate decreased as social jetlag increased. Multiple logistic regression analysis showed that the adjusted odds ratio (95% confidence interval) of CKD for 1–2 h of social jetlag was 0.926 (0.660–1.299), while the odds ratio for more than 2 h was 2.042 (1.328–3.139) when less than 1 h was used as reference. This study found that social jetlag and risk of CKD were significantly related in the Korean working population.

## Introduction

Chronic kidney disease (CKD) confers a heavy burden on societies and healthcare systems worldwide because of the substantial associated morbidity that can lead to end-stage renal disease and is one of the leading causes of death^1^. Thus, prevention of CKD and identification of risk factors are important issues in public health^2^. Hypertension, diabetes, and glomerulonephritis are well-known risk factors for CKD^1^. Moreover, occupational and environmental exposure to heavy metals and pesticides are associated with CKD^3–5^. Working in hot ambient conditions and dehydration may decrease kidney function^6^. Furthermore, recent research has revealed that shift work and long work hours are associated with CKD^7,8^. Sleep deprivation and circadian misalignment have been suggested as possible explanations for the abovementioned association. In addition, social jetlag may cause sleep deprivation and circadian misalignment.

Social jetlag refers to the discrepancy between social time and the body’s internal rhythm^9^. The key feature of social jetlag is the ‘difference in the sleep–wake cycle between work (school) days and free days^9,10^. Some proportions of working populations have this discrepancy, and the prevalence of social jetlag is higher among persons with the evening chronotype in the general daytime working situations^11^. Social jetlag is a form of circadian misalignment, which can occur in shiftwork, jetlag due to long-distance air travel, and daylight saving time in summers^12^. Circadian misalignments can lead to unfavorable health outcomes, including fatigue, sleep disturbance, cardiometabolic disease, and mental health problems^12–15^. Similar to, other types of circadian misalignment, the linking between social jetlag and unfavorable health outcomes, including sleep disturbance, depression, and obesity, has been revealed in earlier studies^9,16–18^.

However, to the knowledge of authors, no study has directly explored the relation between social jetlag and CKD. Thus, with this study, we aim to investigate the relation between social jetlag and CKD.

## Methods

### Data collection and participants

**Figure 1:**
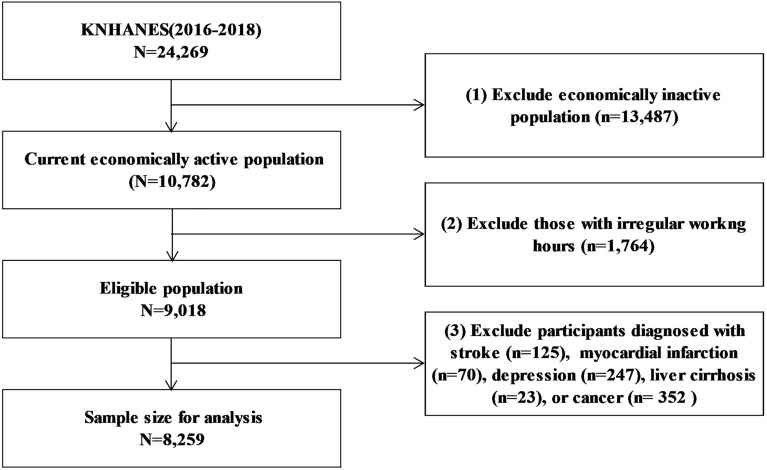
Schematic diagram depicting the selection of the study population.

This study used data from the Korea National Health and Nutrition Examination Survey (KNHANES), which comprises a series of cross-sectional, nationally representative, population-based surveys on the health and nutritional status of Korean citizens, that is conducted by the Korea Centers for Disease Control and Prevention^19^. The representativeness of the KNHANES is based on multistage cluster probability sampling, stratified by geographic region, gender, and age. We used anonymized KNHANES data phase VII from 2016 to 2018, wherein only sleep time on weekends and weekdays was separately investigated. Of the 24,269 individuals who participated in the surveys (8150 in 2016, 8127 in 2017, and 7992 in 2018), we included only the currently economically active population (N = 10,782). After excluding individuals with irregular work hours, such as those with part-time jobs and those who work shifts (n = 1764), 9018 participants were eligible for study participation. The final sample for analysis included 8259 participants, after further exclusion of participants who were diagnosed with diseases that are related to renal function: stroke (n = 125), myocardial infarction (n = 70), depression (n = 247), liver cirrhosis (n = 23), cancer (n = 352) (Fig. 1).

### Ethics approval

At the time of the KNHANES survey, citizens were notified that their households had been randomly selected for voluntarily participating in the nationally representative survey that was conducted by the Ministry of Health and Welfare of Korea. All the KNHANES participants provided written informed consent. The study was conducted in accordance with the guidelines of the Declaration of Helsinki. Approval of the KNHANES by an institutional review board was considered unnecessary in 2015, according to the newly revised Bioethics and Safety Act Article 2 and Enforcement of the Bioethics and Safety Act Article 2-2. The present study protocol was exempted from deliberation by the Institutional Review Board of Seoul St. Mary’s Hospital, Catholic University of Korea (study number: KC20ZISI0555).

### Study variables

The KNHANES collected information on the participants’ socioeconomic status, and participants underwent anthropometric measurements, health interviews including sleep behaviors, physical examinations, and nutritional surveys through face-to-face interviews.

### Social jetlag

Social jetlag conditions were measured using questions about typical sleep-onset and wake times on weekdays and weekends. In addition, the midpoint of sleep was evaluated as the midpoint of the sleep start time (adding sleep latency time before sleep) and the wake time.

The midpoint of sleep time was then calculated separately for the weekdays (midpoint of sleep time on a weekday [MSW]) and weekend or free days (midpoint of sleep time on free days [MSF]). According to the formula established by Wittman et al.^10^, social jetlag was estimated as the absolute value of the difference (in hours) between the midpoints of sleep times on weekdays and weekends (MSF-MSW). In this study, the social jetlag of 98% of the participants was between − 0.75 and 3.75 h. Therefore, we classified the participants into the following three groups: less than + 1 h, + 1 to less than + 2 h, and more than + 2 h of social jetlag. The reference category was less than 1 h, as reported previously^20,21^.

### Assessment of kidney function

In fasting blood samples (12-h overnight), obtained through antecubital venipuncture, the serum creatinine level was measured using the Hitachi Automatic Analyzer 7600 (Hitachi, Tokyo, Japan) and CREA reagent (Roche Diagnostics, Mannheim, Germany). We calculated the estimated glomerular filtration rate (eGFR) by using the serum creatinine value according to the Chronic Kidney Disease Epidemiology Collaboration (CKD-EPI) equation that, across a broad range of populations, more accurately categorizes the risk for mortality and ESRD than does the Modification of Diet in Renal Disease (MDRD) study equation^22^. Participants with an eGFR less than 60 ml/min/1.73 m^2^ were defined as CKD case^23^.

### Other variables

Household income was used to measure income levels because they could be an indicator of an individual’s material resource status, especially in women who may not be the primary household income earners^24^. Therefore, we used the equivalized household income (EHI), which was calculated as the gross household income divided by the square root of household size. Accordingly, the participants were classified into four groups (low, low-middle, middle, and high). Marital status was classified into three groups: married, unmarried, and other (about to be wedded, separated, or divorced). We used additional variables, such as education level (elementary school, middle school, high school, and ≥ college), occupation (white collar, blue collar, pink collar, or others), smoking habits (non-smoker, current smoker, or ex-smoker), drinking problem (yes or no), and self-rated health (fair or poor). The underlying diseases, such as hypertension and diabetes, were determined based on the self-reported information of the disease that had been diagnosed by doctors.

### Statistical analysis

The proportions of the general characteristics of the study population are presented in accordance with the social jetlag categories. Multiple logistic regression analysis was used to calculate the odds ratio (OR) and 95% confidence interval (CI) for CKD in the 1–2 h or > 2 h social jetlag groups compared to that in the reference group (< 1 h), after adjusting for demographic factors. Model 1 was constructed as a crude model and was not adjusted for any variable. Model 2 was adjusted for age and sex. Model 3 was further adjusted for marital status, occupation, weekly work hours, smoking, drinking, and self-rated health in addition to the factors in Model 2. The association between social jetlag and constant eGFR was evaluated using a generalized additive model and nonparametric regression analysis for ascertaining nonlinear relationships, after adjusting for age, sex, marital status, occupation, weekly work hours, smoking, drinking, hypertension, diabetes, obesity, and self-rated health. In this model, a smoothing spline function with 5 degrees of freedom was used. The test to determine if the basis dimension for a smooth is acceptable was based on estimating the residual variance by getting the difference between residuals that are close neighbors according to the smooth’s (numerical) variables^25^. Finally, a stratified analysis was conducted according to sex, age groups, education level, marital status, occupation, EHI, weekly work hours, hypertension, diabetes, and self-rated health categories. Data were analyzed using SAS software (version 9.4; SAS Institute Inc., Cary, NC, USA). Figures were drawn using GAM package of R software (version 3.4.4; R Foundation for Statistical Computing, Vienna, Austria). Two-tailed *p* values less than 0.05 were considered statistically significant.

## Results

The characteristics of the participants according to their social jetlag categories are presented in Table 1. The proportion of those with < 1, 1–2, and > 2 h of social jetlag was 63.80, 25.67, and 10.53%, respectively. The prevalence of 2 h of social jetlag was higher in those who are of younger age, are unmarried, highly educated, current smokers or problem drinkers than others.

**Table 1:** Participant characteristics stratified according to the categories of social jetlag.

	n	Social jetlag		
		< 1 h	1 to < 2 h	≥ 2 h
Age				
< 30	904 (10.95)	304 (33.63)	265 (29.31)	335 (37.06)
30–39	1661 (20.11)	849 (51.11)	596 (35.88)	216 (13.00)
40–49	1991 (24.11)	1158 (58.16)	657 (33.00)	176 (8.84)
50–59	1897 (22.97)	1401 (73.85)	389 (20.51)	107 (5.64)
≥ 60	1806 (21.87)	1557 (86.21)	213 (11.79)	36 (1.99)
Sex				
Male	4380 (53.03)	2893 (66.05)	1035 (23.63)	452 (10.32)
Female	3879 (46.97)	2376 (61.25)	1085 (27.97)	418 (10.78)
Marital status				
Married	6063 (73.41)	4127 (68.07)	1566 (25.83)	370 (6.10)
Never married	1429 (17.30)	548 (38.35)	436 (30.51)	445 (31.14)
Others^a^	767 (9.29)	594 (77.44)	118 (15.38)	55 (7.17)
Education (missing = 1)				
Elementary	1118 (13.54)	957 (85.60)	130 (11.63)	31 (2.77)
Middle	787 (9.53)	608 (77.26)	118 (14.99)	61 (7.75)
High	2563 (31.04)	1614 (62.97)	638 (24.89)	311 (12.13)
≥ College	3790 (45.89)	2089 (55.12)	1234 (32.56)	467 (12.32)
Occupation (missing = 2)				
White collar	3628 (43.94)	1982 (54.63)	1199 (33.05)	447 (12.32)
Pink collar	1544 (18.70)	1062 (68.78)	338 (21.89)	144 (9.33)
Blue collar	2454 (29.72)	1670 (68.05)	522 (21.27)	262 (10.68)
Others^b^	630 (7.63)	554 (87.94)	59 (9.37)	17 (2.70)
Equivalized household income^c^ (missing = 10)				
Low	868 (10.52)	802 (80.88)	115 (13.25)	51 (5.88)
Low-medium	1859 (22.54)	1233 (66.33)	439 (23.61)	187 (10.06)
Medium–high	2546 (30.86)	1568 (61.59)	674 (26.47)	304 (11.94)
High	2976 (36.08)	1757 (59.04)	892 (29.97)	327 (10.99)
Weekly working hours				
≤ 51	6608 (80.01)	4151 (62.82)	1760 (26.63)	697 (10.55)
≥ 52	1651 (19.99)	1118 (67.72)	360 (21.80)	173 (10.48)
Smoking (missing = 56)				
Non-smoker	4644 (56.61)	2907 (62.60)	1285 (27.67)	452 (9.73)
Current smoker	1850 (22.55)	1091 (58.97)	466 (25.19)	293 (15.84)
Ex-smoker	1709 (20.83)	1248 (73.03)	360 (21.06)	101 (5.91)
Problem drinking (missing = 12)				
No	6979 (84.62)	4476 (64.14)	1797 (25.75)	706 (10.12)
Yes	1268 (15.38)	784 (61.83)	323 (25.47)	161 (12.70)
Hypertension (missing = 40)				
No	6705 (81.18)	4062 (60.58)	1858 (27.71)	785 (11.71)
Yes	1514 (18.44)	1197 (79.06)	253 (16.71)	64 (4.23)
Diabetes				
No	7707 (93.32)	4826 (62.62)	2036 (26.42)	845 (10.96)
Yes	552 (6.68)	443 (80.25)	84 (15.22)	25 (4.53)
Obesity				
No	5335 (64.60)	3306 (61.97)	1436 (26.92)	593 (11.12)
Yes	2924 (35.40)	1963 (67.13)	684 (23.39)	277 (9.47)
Self-rated health				
Fair	7162 (86.72)	4543 (63.43)	1870 (26.11)	749 (10.46)
Poor	1097 (13.28)	726 (66.18)	250 (22.79)	121 (11.03)
Total	8259 (100)	5269 (63.80)	2120 (25.67)	870 (10.53)

^a^Widowed, separated, or divorced.

^b^Workers in agriculture, forestry, fishery and military service.

^c^Gross household income was divided by square root of household size.

**Figure 2:**
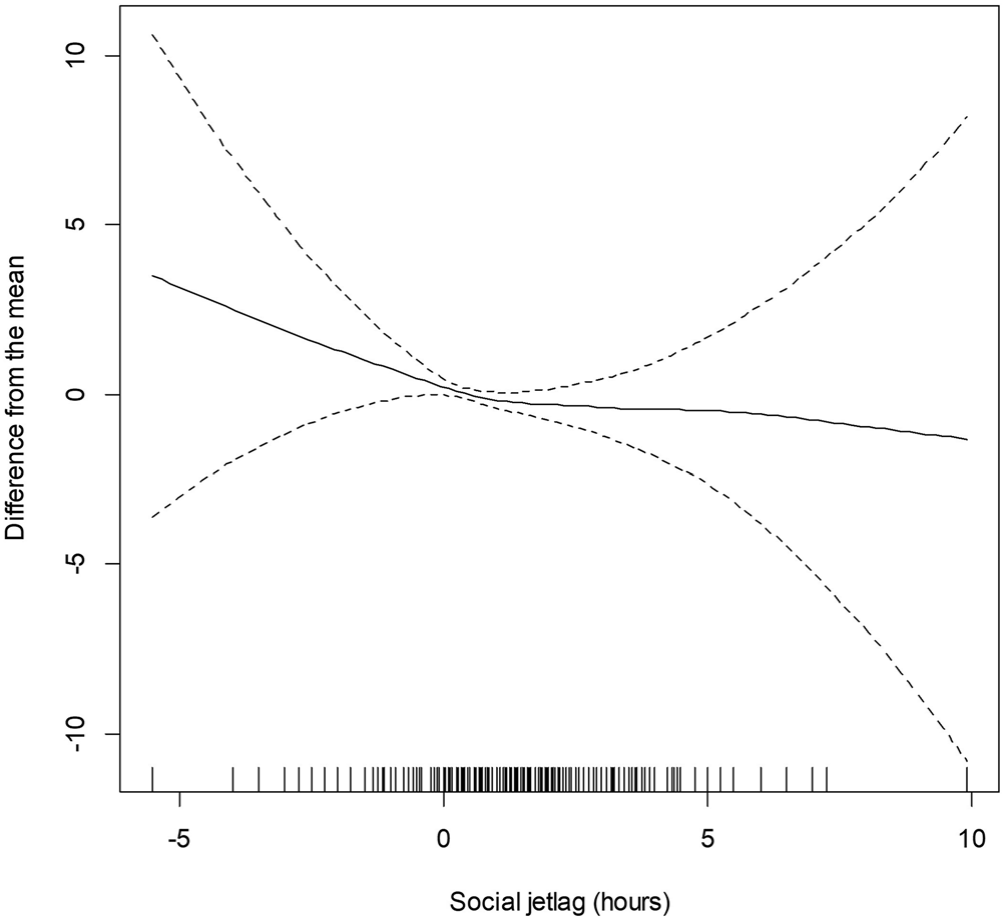
Nonparametric association between social jetlag (hours) and standardized estimated glomerular filtration rate (mL/min/1.73 m^2^) that was adjusted for age, sex, marital status, occupation, weekly working hours, smoking, drinking, hypertension, diabetes, obesity, and self-rated health. Note that the y-axis is "difference from the mean", not the actual value of estimated glomerular filtration rate.

As shown in Fig. 2, the result of GAM with smoothing spline function indicated that the eGFR decreased as social jetlag increased when the difference in the midpoint of sleep times between weekdays and weekends (MSF − MSW) was a positive value. A total of 338 (4.09%) participants were identified as CKD. The Crude ORs of CKD among the 1–2 h and > 2 h social jetlag groups in Model 1, with the < 1 h group as a reference, were 0.543 (95% CI = 0.395–0.749) and 0.886 (95% CI = 0.603–1.300), respectively (Table 2). However, this association was significantly reversed in the model with analyses adjusted for age and sex. The ORs for the groups with 1–2 h and > 2 h social jetlag were 0.908 (95% CI 0.649–1.268), and 2.234 (95% CI 1.473–3.387) in Model 2, respectively. In the model that was further adjusted for marital status, occupation, weekly work hours, smoking, drinking, hypertension, diabetes, and self-rated health, the ORs were significantly higher among the groups with social jetlag of > 2 h than the group with social jetlag of < 1 h (OR = 2.042, 95% CI 1.328–3.139).

**Table 2:** Risk of chronic kidney disease according to the categories of social jetlag.

Social jetlag	n (%)	Model 1^a^	Model 2^b^	Model 3^c^
< 1 h	211/5269 (2.55)	1 (reference)	1 (reference)	1 (reference)
1 to < 2 h	47/2120 (0.57)	0.543 (0.395–0.749)	0.908 (0.649–1.268)	0.926 (0.660–1.299)
≥ 2 h	31/870 (0.38)	0.886 (0.603–1.300)	2.234 (1.473–3.387)	2.042 (1.328–3.139)

^a^Crude odds ratio was calculated by logistic regression.

^b^Adjusted odds ratio was calculated by multiple logistic regression analysis after adjusting for age and sex.

^c^Adjusted odds ratio was calculated by multiple logistic regression analysis after adjusting for age, sex, marital status, occupation, weekly working hours, smoking, drinking, hypertension, diabetes, obesity, and self-rated health.

In the subgroup analysis (Table 3), male sex, the highest income groups, those who did not work long hours, those without diabetes, and those with obesity or poor self-rated health showed relatively higher OR of CKD related with social jetlag, and there were dose–response relationships in these subgroups. However, some associations were not significant.

**Table 3:** The risk of chronic kidney disease according to the categories of social jetlag by subgroup^a^.

	Social jetlag			*p* for trend
	< 1 h	1 to < 2 h	≥ 2 h	
Subgroup by age				
< 30	1 (reference)	0.677 (0.252–1.815)	0.658 (0.243–1.780)	0.385
30–39	1 (reference)	0.955 (0.415–2.199)	2.031 (0.795–5.190)	0.226
40–49	1 (reference)	1.264 (0.509–3.140)	2.062 (0.637–6.668)	0.247
50–59	1 (reference)	1.777 (0.891–3.546)	3.038 (1.113–8.293)	0.014
≥ 60	1 (reference)	0.523 (0.257–1.061)	2.559 (0.9834–6.661)	0.957
Subgroup by sex				
Male	1 (reference)	1.215 (0.737–2.003)	3.175 (1.727–5.835)	0.001
Female	1 (reference)	0.726 (0.457–1.153)	1.317 (0.710–2.445)	0.974
Subgroup by marital status				
Married	1 (reference)	0.924 (0.615–1.387)	2.169 (1.230–3.827)	0.103
Never married	1 (reference)	1.108 (0.479–2.564)	1.254 (0.531–2.960)	0.607
Others^b^	1 (reference)	0.765 (0.254–2.298)	3.321 (0.905–12.185)	0.277
Subgroup by education				
Elementary	1 (reference)	0.593 (0.228–1.542)	4.885 (1.603–14.886)	0.224
Middle	1 (reference)	1.113 (0.407–3.044)	2.510 (0.780–8.079)	0.185
High	1 (reference)	0.912 (0.454–1.829)	1.949 (0.843–4.506)	0.274
≥ College	1 (reference)	1.024 (0.623–1.686)	1.362 (0.696–2.665)	0.454
Subgroup by occupation				
White collar	1 (reference)	0.846 (0.493–1.452)	1.971 (1.046–3.714)	0.141
Pink collar	1 (reference)	0.968 (0.426–2.197)	1.333 (0.414–4.292)	0.746
Blue collar	1 (reference)	1.003 (0.565–1.781)	1.645 (0.745–3.633)	0.353
Others^c^	1 (reference)	0.399 (0.085–1.861)	3.020 (0.286–31.937)	0.676
Subgroup by equivalized household income^d^				
Low	1 (reference)	0.796 (0.319–1.988)	3.030 (0.898–10.220)	0.331
Low-medium	1 (reference)	0.668 (0.318–1.405)	2.720 (1.175–6.298)	0.251
Medium–high	1 (reference)	0.805 (0.398–1.627)	0.772 (0.285–2.095)	0.498
High	1 (reference)	1.213 (0.693–2.123)	2.306 (1.134–4.688)	0.037
Subgroup by weekly working hours				
≤ 51	1 (reference)	1.016 (0.703–1.469)	2.104 (1.289–3.435)	0.024
≥ 52	1 (reference)	0.592 (0.243–1.440)	1.829 (0.747–4.480)	0.597
Subgroup by hypertension				
No	1 (reference)	0.975 (0.653–1.455)	1.735 (1.054–2.854)	0.098
Yes	1 (reference)	0.766 (0.393–1.493)	2.971 (1.197–7.3720)	0.267
Subgroup by diabetes				
No	1 (reference)	0.956 (0.665–1.372)	1.978 (1.254–3.120)	0.033
Yes	1 (reference)	0.873 (0.316–2.413)	1.659 (0.407–6.761)	0.702
Subgroup by obesity				
No	1 (reference)	0.942 (0.632–1.405)	1.404 (0.780–2.525)	0.501
Yes	1 (reference)	0.849 (0.444–1.623)	3.846 (2.002–7.390)	0.003
Subgroup by self-rated health				
Fair	1 (reference)	0.827 (0.564–1.213)	1.896 (1.187–3.027)	0.116
Poor	1 (reference)	1.607 (0.761–3.393)	3.262 (1.058–10.052)	0.031

^a^Adjusted odds ratio was calculated by multiple logistic regression analysis after adjusting for age, sex, marital status, occupation, weekly working hours, smoking, drinking, hypertension, diabetes, obesity, and self-rated health.

^b^Widowed, separated, or divorced.

^c^Workers in the agriculture, forestry, fishery, and military sectors.

^d^Gross household income was divided by the square root of household size.

## Discussion

This study observed the linking between social jetlag and the CKD, particularly among middle-aged or older workers. Although statistically significant association between social jetlag and CKD was not determined in unadjusted models, the association between social jetlag > 2 h and CKD was observed in the adjusted models 1 and 2. Model 2 included sex and age, whereas Model 3 additionally included occupation, marital status, weekly working hours, smoking, drinking, hypertension, diabetes, and self-rated health. This may be due to the fact that, despite social jetlag being more prevalent among younger workers, its deleterious impact on the kidney is more pronounced among workers in their fifties. As seen in subgroup analysis, social jetlag showed greater association with CKD among certain subgroups of workers (e.g., those not working long hours, those without diabetes, those with obesity, and those with poor self-rated health).

Several plausible mechanisms might explain the relationship between social jetlag and CKD. Social jetlag is a form of circadian misalignment^12^, and it may lead to insufficient sleep duration, poor sleep quality, and insufficient recovery^26^. First, circadian misalignment may mainly account for the relationship between social jetlag and CKD. In the suprachiasmatic nucleus(SCN) central circadian clock is located (SCN) and many peripheral organs show circadian rhythmicity^27^. Furthermore, renal function is regulated by the circadian pattern, and includes glomerular filtration, which oscillates in accordance with the circadian clock^28,29^. The circadian clock affects various levels of cellular function, including transcription, translation, and post-translational changes^30,31^. In the present analysis, the eGFR showed decreasing trend in the generalized additive model as the duration of the social jetlag increased. Misalignment of the circadian clock is related to CKD progression in animal models^32^. Shift work, another type of circadian misalignment in a significant proportion of the working population, increases the risk of chronic kidney dysfunction^7,33^.

Another possible contributor may be the insufficient sleep duration of workdays and low sleep quality. Insufficient sleep duration and low sleep quality are closely related to CKD or proteinuria^34–36^. The findings of previous studies showed some similarity with the result of this study, and they provided indirect evidence of findings of this study. Insufficient recovery due to social jetlag may decrease renal function. In addition, long work hours are associated with CKD and can lead to insufficient recoveries, like social jetlag^8,37^. Further explorations are required on mechanisms of social jet lag and health.

The strength of the current study is the use of a nationally representative sample of the Korean working population. However, since this study is a cross-sectional study, the temporal sequence of the exposure and health status and the causal inference cannot be verified. For this reason, there is a possibility of reverse causation, in that CKD may influence sleep and chronotype. The future prospective cohort study can clarify the causal direction between social jetlag and CKD. Another limitation of the present study is the operational definition of CKD. CKD is defined as the existence of structural or functional abnormalities in the kidneys that persist for at least three months^1^. However, due to the nature of the study design, we used only a single measurement of eGFR. This may lead to information bias as well as an overestimation of the prevalence of CKD. Also, other potential confounding, such as various causes of CKD, were not sufficiently considered, except for hypertension and diabetes. Moreover, the KNANES did not investigate information on renal disease morbidity, including glomerulonephritis, and could not exclude them from the study population. For this reason, selection bias may influence the results of the current study. Finally, sleep and wake times were self-reported, which may have resulted in information bias. Objective measurement of sleep time, such as actigraphy can reduce measurement errors by using subjective reports of sleep time.

In conclusion, social jetlag was associated with reduced eGFR among the general working population in Korea. To reduce CKD in the working population, workplace interventions may be needed to reduce the influence of social jetlag. For example, it is possible that a work schedule that employees have some level of control over, as well as working hours that may reflect an individual’s chronotype, might be effective measures in preventing social jetlag. In the future, more studies on social jetlag and health are required for better understanding and prevention of this condition. Prospective cohort and interventional studies are necessary to clarify the causal relationship between the two and to develop effective preventive measures.

## Data Availability

The data for this study were accessed through the KNHANES homepage (https://knhanes.kdca.go.kr/knhanes/eng/index.do).
